# Bibliometric analysis of bone metastases from lung cancer research from 2004 to 2023

**DOI:** 10.3389/fonc.2024.1439209

**Published:** 2024-08-06

**Authors:** Jing Tang, Zhangui Gu, Zongqiang Yang, Long Ma, Qiang Liu, Jiandang Shi, Ningkui Niu, Yanyang Wang

**Affiliations:** ^1^ Department of Radiotherapy, General Hospital of Ningxia Medical University, Yinchuan, China; ^2^ Department of Orthopedic, General Hospital of Ningxia Medical University, Yinchuan, China; ^3^ First Clinical Medical College, General Hospital of Ningxia Medical University, Yinchuan, China

**Keywords:** lung cancer, bone metastasis, bibliometrics, current state, research

## Abstract

**Background:**

Bone metastases of lung cancer (BMLC) severely diminish patients’ quality of life due to bone-related events, and the lack of clear guidelines globally regarding medical and surgical treatment significantly reduces patient survival. While knowledge about BMLC has grown exponentially over the past two decades, a comprehensive and objective bibliometric analysis remains absent.

**Methods:**

A comprehensive bibliometric analysis was conducted on relevant literature on BMLC extracted from the Web of Science database from 2004 to 2023 by Biblioshiny, VOSviewer, Scimago Graphica, CiteSpace, and Microsoft Office Excel Professional Plus 2016 software. 936 papers related to BMLC were extracted from the Web of Science Core Collection (WoSCC). The number of publications, countries, institutions, global collaborations, authors, journals, keywords, thematic trends, and cited references were then visualized. Finally, the research status and development direction in the last 20 years were analyzed.

**Results:**

This study included a total of 936 papers on BMLC from 2004 to 2023. There has been a steady increase in global publications each year, peaking in 2021. China had the highest number of publications, followed by Japan and the United States. Additionally, China had the most citations with an H-index of 35, while the US followed with an H-index of 34, highlighting their significant contributions to the field. “Frontiers in Oncology” had the highest number of publications. CiteSpace analysis identified “lung cancer,” “bone metastasis,” and “survival” as the top high-frequency keywords, encapsulating the core research focus. Keyword clustering analysis revealed six main clusters representing the primary research directions. Burst analysis of keywords showed that “skeletal complications” had the highest burst intensity from 2005 to 2013, while recent research trends include “immunotherapy” and “denosumab,” with bursts from 2021 to 2023. Trend topic analysis indicated that “non-small cell lung cancer,” “immunotherapy,” and “immune checkpoint inhibitors” represent the cutting-edge research directions in this field.

**Conclusion:**

This article reveals the current status and trend of research on BMLC, which is increasing worldwide. China and the United States have contributed the most, but international cooperative research on BMLC should be strengthened. The pathogenesis, early prevention, and individualized treatment of BMLC need to be strengthened for further study, and immunotherapy is the next hotspot of lung cancer bone metastasis research.

## 1 Introduction

Lung cancer is the malignant tumor with the highest rate of morbidity and mortality worldwide today and represents a severe threat to the life and health of human beings. According to the American Cancer Society’s Global Cancer Statistics Report 2022, lung cancer is both the most common type of cancer worldwide and the leading cause of death among people living with cancer (1). Lung cancer has an insidious onset and is challenging to diagnose at an early stage. Approximately 50% of lung cancers are advanced (stage IV) at diagnosis, and bone metastasis is one of the significant sites of hematogenous metastasis (2). With the progress and development of precision medicine technology, there has been a gradual improvement in the 5-year survival rate for patients with advanced lung cancer (3, 4). The prolonged survival of patients is accompanied by an increased risk of bone metastasis and skeletal-related events (SREs) (5, 6).

BMLC often causes SREs, such as bone pain, pathologic fracture, spinal cord compression or paraplegia, hypercalcemia, and pain caused by related treatments. These SREs seriously affect patients’ quality of life and shorten patients’ survival. The favorable sites of lung cancer bone metastasis are in the proximal part of the spine and trunk bone, and those occurring in the spine account for 50%, the femur account for 25%, and the ribs and sternum account for 12% (7–9). Studies have shown that the survival time of patients with BMLC can be reduced by half once SREs occur (10). Patient survival is further shortened when combined with severe SREs such as hypercalcemia, pathologic fracture, spinal cord compression, and other complications (11, 12). Therefore, active prevention and treatment of bone metastases, as well as effective control of the primary disease, are of paramount importance. However, the current research literature and data on the pathogenesis, diagnosis, and treatment of BMLC are scattered, which could be more conducive to relevant researchers efficiently grasping the main results of previous studies and refining the hotspots for future prospective studies.

Although Meta-analysis and traditional reviews have conducted relevant studies on BMLC, they only focus on specific perspectives or indicators, with a narrower scope of research design and more limited analysis and discussion. Bibliometrics focuses on the quantification of a complete body of knowledge. By analyzing the existing quantitative literature, it is possible to use intuitive maps to predict the future direction of a research field and systematically analyze the research progress of various countries, institutions, authors, and disciplines. Only a few scientists have provided a comprehensive and quantitative estimate of the scientific output of bone metastasis’s performance in lung cancer through bibliometrics. Therefore, this paper aims to comprehensively and objectively analyze the indicators of the documents related to lung cancer bone metastasis through bibliometrics to elucidate the trends, hotspots, and directions of the research related to lung cancer bone metastasis to the researchers and also to provide new clues and ideas for the future study, diagnosis, and treatment of lung cancer bone metastasis.

## 2 Materials and methods

### 2.1 Data collection

The literature extracted from the Science Citation Index Expanded in the Web of Science Core Collection (WosCC) database compiles comprehensive citation and publication information across various scientific fields.

### 2.2 Search strategies

**Figure 1:**
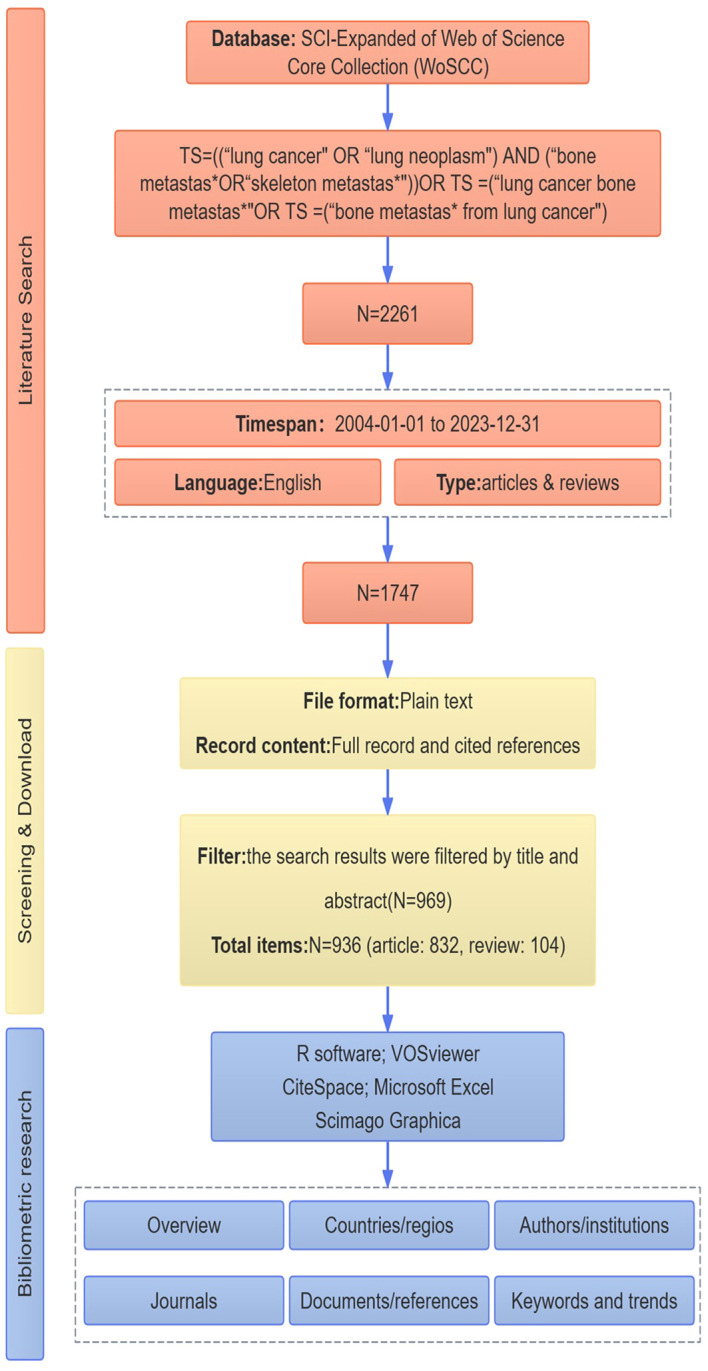
Flowchart of the study.

All published papers were collected from WoSCC, and two investigators identified relevant published between January 01, 2004, and December 31, 2023, using the following search strategy: TS = ((“lung cancer” OR “lung neoplasm”) AND (“bone metastas*” OR “skeleton metastas*”)) OR TS = (“lung cancer bone metastas*”) OR TS = (“bone metastas* from lung cancer”) AND publishing year = (2004–2023) AND document types = (articles & reviews) AND language = (English). After the preliminary data retrieval, two researchers (ZG Gu and ZQ Yang) screened all manuscripts separately to ensure they were relevant to the theme of this study, with any discrepancies being resolved by the experienced corresponding author (JD Shi, NK Niu, YY Wang). The flowchart of the study is shown in **Figure 1**.

All the records of publications, including year of publication, title, author names, affiliations, countries/regions, abstract, keywords, and name of publishing journals, The included records were exported and saved as plain text files with “Full Record and Cited References” for further bibliometric analysis.

### 2.3 Bibliometric analysis

Our bibliometric analysis followed a general to detailed path, including an overview of countries/regions, institutions/authors, journal distribution, documents/references, keywords, and trends. The collected data were later imported into Biblioshiny (R version 4.3.3), VOSviewer (version 1.6.20), Scimago Graphica (Version 1.0.41), CiteSpace (version 6.3.R1), and Microsoft Office Excel Professional Plus 2019 (Redmond WA, USA) for further analysis.

## 3 Results

### 3.1 Overall publication of global literature

**Figure 2:**
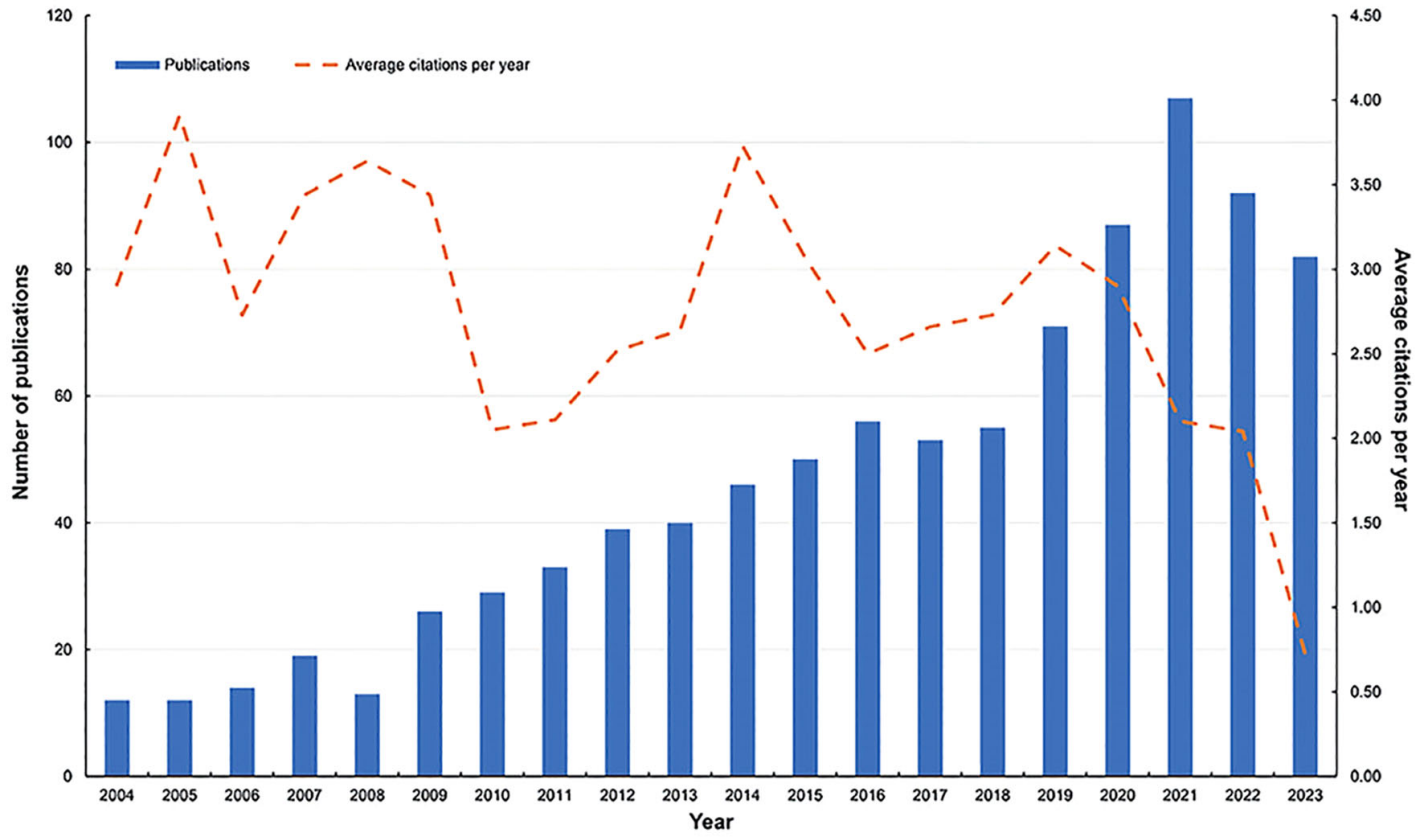
Annual output of publications and average yearly article on BMLC.

The flowchart of the study is shown in **Figure 1**. This study included 936 BMLC articles, 832 articles, and 104 reviews. The annual number of publications (NP) related to BMLC is shown in **Figure 2**. Annual publications increased from 12 in 2004 to 82 in 2023. However, there is an upward trend, peaking in 2021 (107 articles).

### 3.2 Analysis of countries/regions and institutions

**Figure 3:**
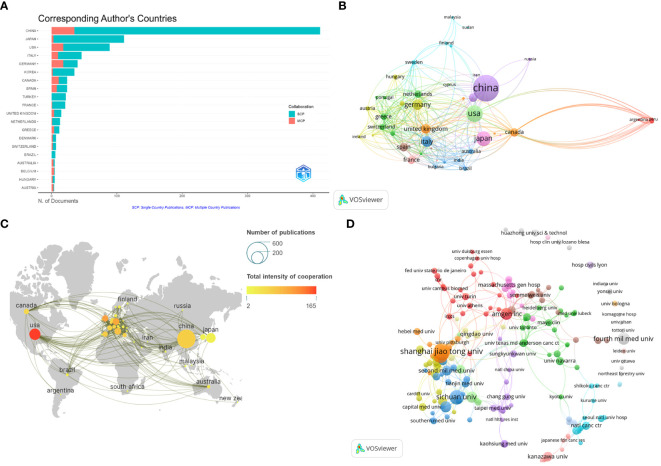
Visualize and analyze active countries and institutions. **(A)** Top 20 corresponding author’s countries;**(B)** The co-authorship map of countries/regions involved in BMLC; **(C)** Country cooperation map; **(D)** The co-authorship map of institutions engaged in BMLC research.

The number of publications in each country was analyzed to identify the countries/regions that have been the major contributors to the field. Researchers from 1543 institutions in 58 countries participated in the study of BMLC. The ten most important countries/regions for production were analyzed according to the country of residence of the corresponding author (**Table 1**). China published the highest number of articles (411, 43.91%). This was followed by Japan (111, 11.86%) and the United States (89, 9.51%) (**Figure 3A**). In addition, China ranked first among all participating countries/regions with 5,387 citations and an H-index of 35. The United States ranks second with 4,439 citations and an H-index of 34. This shows that China and the United States are the most significant contributors to the field. The total number of publications from these two countries exceeds half of the total. The 58 countries are divided into 16 clusters. The country of authorship in the articles and the circle size in the figure represent the number of publications (**Figure 3B**). The geographic map of the number of publications and the intensity of cooperation of each country is shown in (**Figure 3C**), and the United States is the country that initiates and participates in the most international collaborations. **Figure 3D** shows a network of collaborative relationships between leading institutions (with at least three publications). **Table 2** shows the top 10 universities with the highest number of published papers, of which nine are from China, and one is from the United States. Shanghai Jiao Tong University published the most articles (38, 4.06%), followed by Fudan University (28, 3.00%) and Sichuan University (24, 2.56%). Among the top ten organizations regarding the number of articles published, Amgen Inc. ranked first with Average citations of 54.00, followed by Second Mil Med univ (19.75) and Shanghai Jiao Tong University (17.16).

**Table 1:** Top 10 most productive countries/regions in BMLC research.

Rank	Country/region	No. (%)	Total citations	Average citations	H-index
1	China	411(43.91)	5387	13.11	35
2	Japan	111(11.86)	2655	23.92	25
3	USA	89(9.51)	4439	49.88	34
4	Italy	46(4.91)	1510	32.83	21
5	Germany	40(4.27)	805	20.13	17
6	Korea	35(3.74)	704	20.11	15
7	Canada	24(2.56)	1652	68.83	15
8	Spain	24(2.56)	615	25.63	14
9	France	21(2.24)	332	16.60	9
10	Turkey	21(2.24)	273	12.41	10

**Table 2:** Top 10 most productive institutions in BMLC research.

Rank	Institution	Country/region	No. (%)	Total citations	Average citations
1	Shanghai Jiao Tong univ	China	38(4.06)	652	17.16
2	Fudan univ	China	28(2.99)	439	15.68
3	Sichuan univ	China	24(2.56)	372	15.50
4	Fourth mil med univ	China	18(1.92)	314	17.44
5	Amgen inc	USA	17(1.82)	918	54.00
6	China med univ	China	15(1.60)	184	12.27
7	Sun yat-sen univ	China	15(1.60)	165	11.00
8	Tongji univ	China	14(1.50)	180	16.36
9	Zhejiang univ	China	13(1.39)	181	13.92
10	Second mil med univ	China	12(1.28)	237	19.75

### 3.3 Authors and co-authors

**Figure 4:**
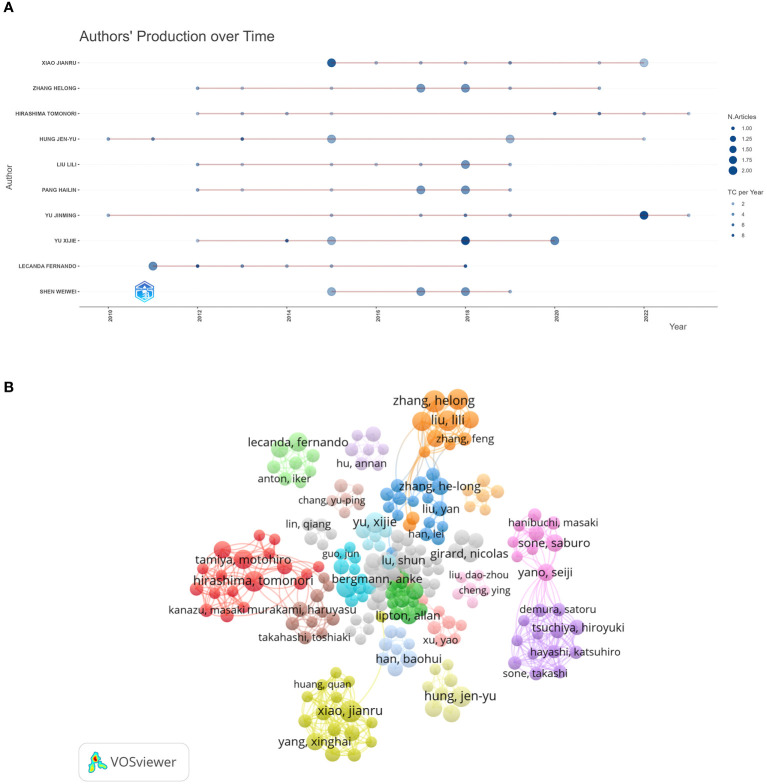
Visualization of active authors analysis. **(A)** Authors’ production over time. **(B)** The visualization map of authors’ co-authorship.

A total of 6,012 authors published research papers on bone metastasis in lung cancer, of which 257 scholars published three or more. 6,012 authors published research papers on BMLC, with 257 authors publishing three or more articles. **Table 3** shows the 10 most published authors in this field. Zhang Helong and Xiao Jianru published the most articles (n = 9), and Lecanda Fernando ranked first in total citations (311) and average citations (44.43). Among the top ten authors, Hong Jenyu and Yu Jinming started their research earlier and continued their contribution to the field in 2022–2023 (**Figure 4A**). A total of 257 authors with at least three publications were grouped into 36 clusters (clusters), and we plotted the authors’ collaborative network graph (co-authors network graph) (**Figure 4B**). A total of 257 authors with at least three publications were divided into 36 clusters. We plotted the collaborative network graph of the authors (co-authors network graph) (**Figure 4B**). Each node represents an author, and larger nodes represent more published papers. Thicker lines indicate closer cooperation between authors. Different colors represent different clusters, and a relatively strong collaboration exists between authors in the same cluster.

**Table 3:** The top 10 authors with the most publications on BMLC.

Rank	Authors	No. (%)	H-index	G-index	Total citations	Average citations
1	Zhang Helong	9(0.96)	7	9	121	13.44
2	Xiao Jianru	9(0.96)	6	9	146	16.22
3	Hung Jen-yu	8(0.85)	6	8	222	27.75
4	Yu Xijie	8(0.85)	7	8	198	24.75
7	Yu Jinming	8(0.85)	6	8	135	16.88
5	Pang Hailin	8(0.85)	7	8	114	14.25
6	Liu Lili	8(0.85)	6	8	104	13.00
8	Hiroshima Tomonori	8(0.85)	5	8	100	12.50
9	Lecanda Ferando	7(0.75)	7	7	311	44.43
10	Shen Weiwei	7(0.75)	7	7	87	12.43

### 3.4 Journal distribution

**Figure 5:**
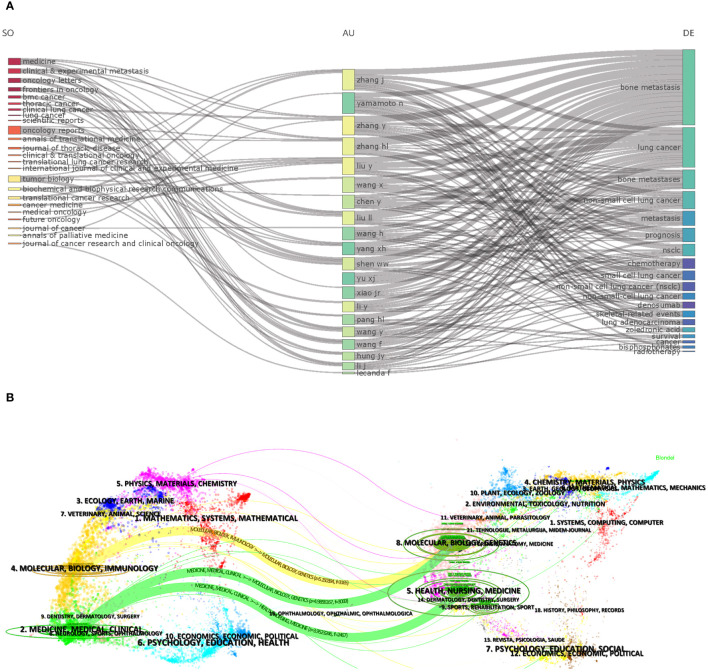
**(A)** Three-field plot (middle field: Authors; left field: Sources; right field: Keywords). **(B)** The dual-map overlay of journals related to LCBM.

A total of 267 journals were involved in publishing articles on bone metastasis research in lung cancer. **Table 4** shows the top 10 journals and citations for BMLC. In addition, the top-ranked journals have high H-indexes and citation counts. This indicates that they play a crucial role in research in the field. Frontiers in Oncology has published the most, followed by Lung Cancer and Oncology Letters (**Table 4**).**Figure 5A** illustrates the authors’ publishing patterns in related topics and journals. Critical research keywords for the top 20 authors included bone metastasis/lung cancer/bone metastases/non-small cell lung cancer/metastasis/prognosis/chemotherapy/small cell lung cancer/denosumab/skeletal-related events/lung adenocarcinoma/zoledronic acid/survival/cancer. Their research is usually published in Medicine, Oncology Reports, and Tumor Biology journals. Among them, Medicine has become the journal that publishes the most articles on bone metastasis of lung cancer. **Figure 5B** shows the dual-map overlay of journals. On the left are the journals that have published articles on BMLC, and on the right is a map of cited journals.

**Table 4:** The top 10 journals with the most publications on BMLC.

Rank	Journal	No. (%)	H-index	G-index	Total citations	IF/JCR	Region
1	Frontiers in Oncology	32(3.42)	9	14	235	4.7/Q2	Switzerland
2	Lung Cancer	29(3.10)	17	29	1679	5.3/Q2	Netherlands
3	Oncology Letters	24(2.56)	9	14	241	2.9/Q3	Greece
4	BMC Cancer	20(2.13)	11	20	455	3.8/Q2	England
5	Medicine	19(2.03)	7	10	117	1.6/Q3	USA
6	Clinical Lung Cancer	18(1.92)	13	18	377	3.6/Q2	USA
7	Clinical & Experimental Metastasis	17(1.82)	13	17	400	4.0/Q2	Netherlands
8	Supportive Care in Cancer	16(1.71)	10	16	355	3.1/Q2	USA
9	Thoracic Cancer	16(1.71)	6	12	145	2.9/Q3	China
10	Cancers	15(1.60)	7	10	122	5.2/Q2	Switzerland

### 3.5 Document citation and reference co-citation

**Figure 6:**
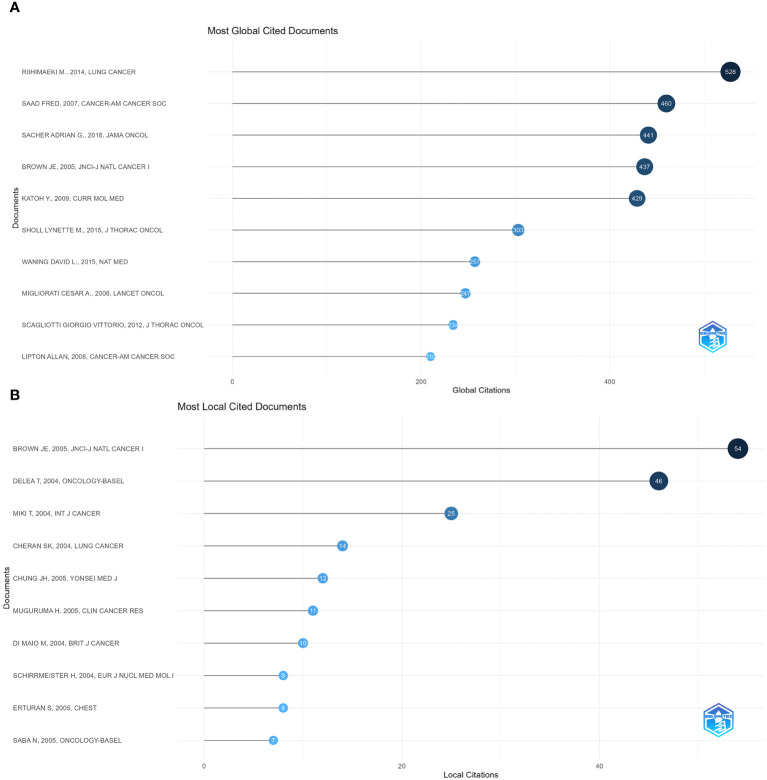
Visualization of document analysis. **(A)** Most global cited documents. **(B)** Most local cited documents.

We ranked the included papers according to their citation data in WoSCC. The top 10 papers were listed according to their number of citations, with the three most cited papers having 528 citations (Pathologic Fractures Correlate With Reduced Survival in Patients With Malignant Bone Disease (13), 460 (Metastatic Sites and Survival in lung cancer (14), and 441 (Prospective Fracture Correlation in Patients With Malignant Bone Disease (15). Metastatic sites and survival in lung cancer pointed out that bone metastasis in lung cancer had the most nervous system (47%) followed by bone (39%). We also assessed the impact of each paper in the field by counting the number of local citations in the current dataset. The ranking of locally cited literature differs slightly from the total cited literature. The highest-ranked paper in the locally cited literature was a study on bone metastases in non-small cell lung cancer by Asuka Tsuya, and the second and third-highest-ranked studies in terms of local citations were both studies on the clinical features, pathophysiology, and therapeutic strategies for metastatic bone disease by author R. E. Coleman. This represents a recognition of the significance and direction of research in this area. **Figures 6A, B** show the ten most cited publications globally and the ten most cited locally.

**Figure 7:**
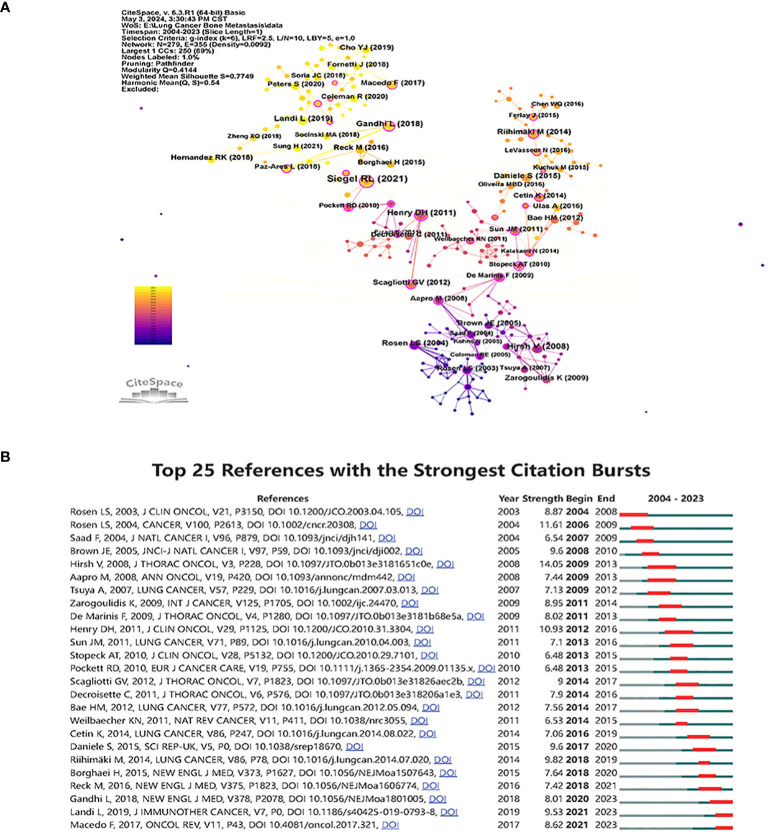
Visualize and analyze references analysis **(A)** Cluster analysis of co-cited references. **(B)** Top 25 references with the strongest citation bursts.

In **Figure 7A**, co-cited references are presented as nodes. The most co-cited reference was “ Cancer statistics, 2019 (16)”, followed by “ Zoledronic acid and survival in patients with metastatic bone disease from lung cancer and elevated markers of osteoclast activity (17)”, “ Randomized, double-blind study of denosumab versus zoledronic acid in the treatment of bone metastases in patients with advanced cancer (excluding breast and prostate cancer) or multiple myeloma (18)” was the third most co-cited article. CiteSpace identified the top 25 references and depicted them in **Figure 7B**, demonstrating the strength and duration of the citation bursts for these references. The top 10 papers are landmarks in studying bone metastasis in lung cancer in order of citation (**Supplementary Table 1**).

### 3.6 Keywords and trends

**Figure 8:**
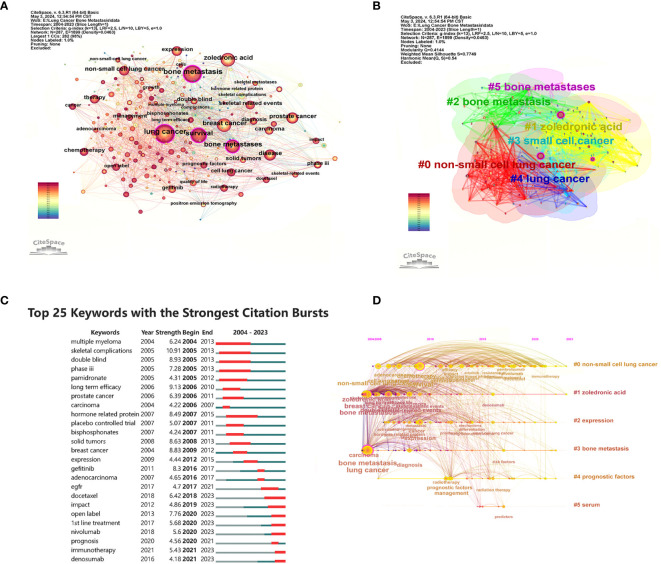
Visualization of keywords analysis. **(A)** The network map of keywords. **(B)** Clusters of keywords; **(C)** Top 25 keywords with the strongest citation bursts. **(D)** Keyword timeline view in LCBM research.

**Figure 9:**
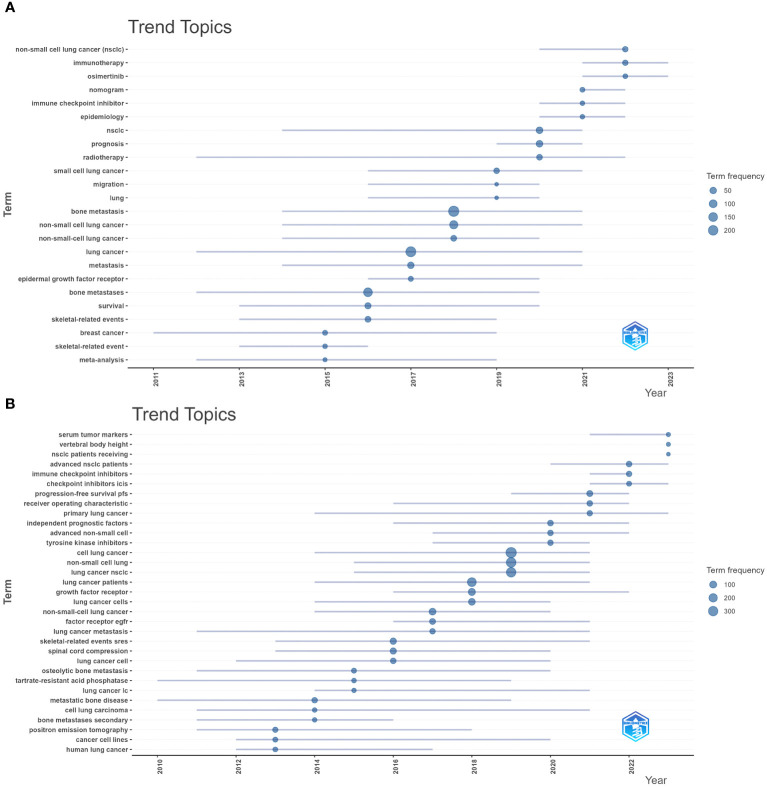
Visualize and analyze Trend Topics **(A)** Author’s keywords (Circle size represents frequency) **(B)**. Visualization of Trend Topics. Abstracts (trigram).

**Figure 8A** shows the high-frequency keywords analyzed by CiteSpace, with “lung cancer,” “bone metastasis,” and “survival” being the top three (larger circles in **Figure 8A**). These keywords summarize the topic. They also represent the research focus of the field. The details of the top 10 keywords are shown in **Supplementary Table 1**. Keyword clustering shows the frequency and distribution of two keywords that occur together. It forms a cluster representing the main research directions in the field (**Figure 8B**). These keywords formed six clusters (Clusters), including “non-small cell lung cancer,” “zoledronic acid,” “small cell cancer,” “lung cancer,” and “bone metastases.” **Figure 8C** shows the top 25 keywords with the strongest keyword bursts, which are intended to reflect research hotspots and trends. Among them, “skeletal complications (2005–2013, intensity 10.91)” was the keyword with the strongest burst intensity. The citation explosion of keywords “immunotherapy” and “denosumab” lasts from 2021 to 2023. From the timeline of keywords regarding treating BMLC, early studies (2005–2012) were focused on phosphonates’ efficacy in solid tumors with bone metastases (**Figure 8D**). Over time, there has been a gradual increase in studies of radiotherapy and chemotherapy (2010–2013). Pembrolizumab, Denosumab, Docetaxel, and nivolumab gradually came into the picture as drugs were developed. The keywords were analyzed by trend topic in Biblioshiny (**Figure 9A**). The top three by frequency of occurrence were “bone metastasis” (247), “lung cancer” (222), and “bone metastases” (147). Biblioshiny analysis of keywords shows that “non-small cell lung cancer” (2020–2022), “immunotherapy” (2021–2023), and “immune checkpoint inhibitors, ICIs” (2020–2022) may represent the frontiers of this research area. The abstracts were analyzed using the trend topic in Biblioshiny (**Figure 9B**). The top three by frequency of occurrence were “cell lung cancer” (395), “non-small cell lung” (318), and “lung cancer nsclc” (304). Biblioshiny analysis of abstracts shows that “serum tumor markers” (2021–2023), “vertebral body height” (2023–2023), and “nsclc patients receiving” (2023–2023) may represent the frontiers of this research area (**Supplementary Table 2**).

## 4 Discussion

Patients with BMLC have an extremely high mortality rate and face serious treatment challenges. Relevant research on BMLC has developed rapidly in recent years. Bibliometrics, as an emerging method, can assess the current status and trend of research in a particular field. Bibliometrics, as a new method, can evaluate the research)) status and trends in a certain field. This paper can provide a reference for researchers to better grasp the development trend and emerging research hotspots through the bibliometric analysis of BMLC-related studies from 2004–2023 in the WoSCC database and the visualization of the international research status in this field.

In this study, we used VOSviewer, CiteSpace, and R to study the research dynamics and hotspots of BMLC in SCI-Expanded. A total of 936 papers were obtained from WoSCC. Between 2004 and 2023, the number of papers published yearly is expected to increase. This is consistent with the increasing trend of new cancers worldwide. The number of new cancer cases worldwide will increase from 18.7 million in 2010 to 23.6 million in 2019 (19). In general, there are fewer publications on BMLC than on solid tumor bone metastases such as breast cancer and lung metastases in prostate cancer (20, 21). This may be because when bone metastases occur in lung cancer, it means that patients have a shorter survival (14), with reported data ranging from 3 to 12 months, depending on the patient’s treatment (22).

China has the most significant publications and has contributed significantly to BMLC research. In addition, nine of the top 10 high-yielding institutions are from China, including Shanghai Jiao Tong University, Fudan University, and Sichuan University. This indicates that LCBM has received increasing attention from Chinese research institutions in recent years. Meanwhile, China ranked first in the H-index. However, the quality of Chinese BMLC research still needs to be improved, as the average number of citations is still at the bottom of the list. The reason for the high number of publications in China may be linked to the high incidence of lung cancer, which continues to have a high incidence (815,000 new lung cancer cases in 2020) and mortality rate. The burden of lung cancer in China is directly related to the high prevalence of smoking, especially among men. The prevalence of smoking among Chinese men, although declining, is still high (50% in 2019) (23). In addition, this has been associated with China’s large population, low rates of early lung cancer screening, and economic development (24). Despite the relatively small number of studies, the United States ranked first in the average number of citations and second in the H-index. This indicates a high level of research quality in the United States. At the same time, the United States plays a central role in the cooperation between countries/regions. Europe and other developed countries cooperate closely, while developing countries cooperate sparsely, as shown in **Figure 3C**.

Therefore, we should strengthen international cooperation to help developing countries. As far as authors are concerned, those experienced in the BMLC have become highly recognized, with Lecanda Fernando being the most cited international author. Among the top two authors with publications in BMLC, Zhang Helong et al. conducted an in-depth study on gene regulation and biomarkers in lung cancer bone metastasis (25–27). Xiao Jianru et al. found that surgery can improve patients’ quality of life with non-small cell lung cancer bone metastasis (28, 29).

Articles of BMLC are most often published in Frontiers in Oncology, followed by Lung Cancer and Oncology Letters. As a high-quality, influential journal in the field, Lung Cancer has the highest impact factor of 5.3 and is the most cited journal. The journals on the list will likely be the significant publication outlets in the field in the future. The most cited paper states that fractures are associated with an increased risk of death in patients with malignant bone disease. Therefore, prevention of fractures is an essential goal of treatment. Zoledronic acid significantly benefits patients with bone metastases by preventing skeletal complications and reducing bone pain. Thus, it allows patients to maintain mobility and functional independence and reduces the risk of death due to fracture complications (13). Nitrogen-containing bisphosphonates inhibit osteoclast-mediated bone destruction and block the release of bone-destroying cytokines and growth factors, thereby retarding the growth of bone tumors. It also inhibits tumor cell functions such as tumor cell adhesion, migration, invasion and proliferation, as well as bone matrix adhesion by inhibiting farnesyl diphosphate synthase (FPPS), thereby inducing apoptosis (30).

In **Figure 8A**, high-frequency keywords reveal the research hotspots of BMLC-related research fields to some extent. The top three high-frequency keywords were “lung cancer,” “bone metastasis,” and “survival.” They represent the most commonly used terms and research hotspots in BMLC research, and “survival” has become a research hotspot in this field. Treatment is the basis for improving survival, and the treatment of lung cancer bone metastasis can be divided into radiotherapy, chemotherapy, targeted therapy, immunotherapy, and multidisciplinary treatment. Radiotherapy can effectively reduce the damage caused by the disease and prolong the life of patients to a certain extent.

In recent years, with the advent of new radiotherapy techniques (such as stereotactic radiotherapy (31) and multidisciplinary oncology management (32), the combination of radiotherapy with new systemic, integrated treatment options (such as targeted therapy (33) and immunotherapy (34) can improve patient’s quality of life to a greater extent, thereby contributing to remission and significantly extending survival—reduction of treatment-related toxicity (35). Adjuvant chemotherapy, surgery, and phosphonate-targeted therapy also improved therapeutic effects (36–38).

The epidermal growth factor receptor tyrosine kinase inhibitors (EGFR-TKI) gefitinib and erlotinib, used in adjuvant chemotherapy, showed significant antitumor activity in lung cancer with EGFR gene mutation (39). It has also been suggested that CT-guided ^125^ I brachytherapy may be a reasonable alternative for relieving painful bone metastases secondary to lung cancer after one cycle of chemotherapy progression. It can accelerate pain relief, improve quality of life, reduce pain recurrence, and reduce cost-effectiveness and similar complications (40), but it needs to be validated in a larger cohort. In recent years, basic research has helped better understand the relevant mechanisms, and targeted therapies are becoming increasingly influential. Receptor activators of the nuclear factor-κB ligand (RANKL)/RANK signaling pathway are critical mediators of bone remodeling. Targeting RANKL with the antibody denosumab is part of the standard treatment for bone loss diseases, including bone metastases (41). However, as with phosphonates, adverse consequences such as hypocalcemia, atypical femur fractures, and jaw osteonecrosis should be noted in clinical practice (42, 43).

Analyze the direction of abstract and keyword trends by Trend Topics. “non-small cell lung cancer,” “immunotherapy,” and “ immune checkpoint inhibitors, ICIs” may herald the frontiers of this research area. Bone metastases occur in more than 50% of patients with non-small cell lung cancer (NSCLC) (44). Adjuvant preoperative chemotherapy is poorly tolerated by patients due to its more pronounced toxicities, with a 5% improvement in 5-year survival (45). The emergence of immune checkpoint inhibitors (ICIs) has dramatically changed the therapeutic outlook for NSCLC patients, and preoperative neoadjuvant immunotherapy regimens have gradually gained attention as an effective and safe treatment option (46). By blocking programmed cell death protein 1 (PD-1)/programmed cell death-ligand 1 (PD-L1) and cytotoxic T lymphocyte-associated antigen-4, PD-L1 and cytotoxic T lymphocyte-associated antigen-4, PD-L1, and cytotoxic T lymphocyte-associated antigen-4, PD-L1 and PD-L1, NSCLC patients have been treated with a new preoperative adjuvant immunotherapy. -associated antigen-4 (CTLA-4) pathways interacting with inhibitory antibodies to enable activated T cells to release cytokines, perforins, granzymes, etc., to kill tumor cells in a rapidly evolving immunotherapeutic regimen (47). Preoperative neoadjuvant immunotherapy and postoperative adjuvant immunotherapy regimens using ICIs have also been investigated as alternatives to preoperative neoadjuvant chemotherapy and postoperative adjuvant chemotherapy regimens. Currently, ICI therapy, either as monotherapy or in combination with platinum-based chemotherapy, is approved as a standard of care for advanced NSCLC (48). Regarding the therapeutic role of ICIs for bone metastases, Asano Y et al. (49) used the PD-1 inhibitors natalizumab and pembrolizumab as well as the PD-L1 inhibitors atezolizumab and durvalumab for the treatment of In patients with advanced bone metastases of NSCLC, the results of the study showed that treatment with ICIs produced favorable therapeutic effects and improved prognosis in patients with advanced bone metastases of NSCLC. After the excellent progress of immunotherapy, many studies have focused on immunotherapy combined with chemotherapy or other drugs in combination regimens. Ma K et al. (50) showed that immunotherapy combined with antiangiogenic therapy is an option for patients with advanced NSCLC with reasonable safety and tolerability and pointed out that bone metastasis is a potential independent negative predictor of median overall survival. However, most of the current studies in this area are phase I and II studies, and in terms of data from phase III studies, the study by Forde P M et al. made significant progress on the regimen of preoperative natalizumab and platinum-containing drug chemotherapy in patients with NSCLC (51). However, experimental data still need to be published because phase III studies of other types of ICIs are still ongoing. More studies are required in order to explore the various kinds of ICIs, their cycles of use, and the choice of LCBM. It is worth proposing that with the development of precision medicine and artificial intelligence, the development of individualized column-line diagrams for BM risk stratification (52–54) and multiple machine learning algorithms provide new research ideas in identifying and predicting the occurrence of bone metastasis and the final clinical outcome of lung cancer (55–59).

## 5 Limitations

This research carries some limitations. Firstly, our study only included the WoSCC database, which may have led to the exclusion of important studies that were not included in this database. Secondly, the exclusion of non-English literature may have an impact on the overall results. Thirdly, our analysis was limited to bibliometric data and did not include a detailed qualitative analysis of the content of the studies, which may provide deeper insights into the topic.

## 6 Conclusion

This study used bibliometrics to reveal lung cancer bone metastasis’s research status and trend. International cooperation in LCBM research should be further strengthened. Our study provides valuable information for understanding and dealing with lung cancer bone metastasis and provides a basis for conducting more in-depth research and searching for more effective treatments. Further research should focus on the pathogenesis, early prevention, and individualized treatment of lung cancer bone metastases to improve patient survival and quality of life. There are some limitations to the study. First, the analyzed data was retrieved only from WoSCC, which may have excluded essential studies not included in that database. Second, only English articles were included in this study, which may have led to the exclusion of some critical studies in non-English language publications. Third, citations can be influenced by outdated research and publication dates. Finally, despite our standardized data processing, the analysis software’s mechanical nature may lead to inaccurate keyword extraction and incomplete article content analysis. However, the impact of the above deficiencies on the overall trend of the article is still within the acceptable range.

## Data availability statement

The original contributions presented in the study are included in the article/**Supplementary Material**. Further inquiries can be directed to the corresponding authors.

## Author contributions

JT: Funding acquisition, Investigation, Methodology, Writing – original draft, Writing – review & editing, Software. ZG: Investigation, Methodology, Software, Writing – original draft, Writing – review & editing. ZY: Investigation, Methodology, Software, Writing – original draft, Writing – review & editing. LM: Writing – review & editing, Methodology. QL: Methodology, Writing – review & editing. JS: Methodology, Supervision, Visualization, Writing – review & editing. NN: Methodology, Supervision, Visualization, Writing – review & editing. YW: Methodology, Supervision, Visualization, Writing – review & editing.
