# Supplementary material for: Bibliometric analysis of bone metastases from lung cancer research from 2004 to 2023
Source: Front Oncol. 2024 Aug 6;14:1439209. doi: 10.3389/fonc.2024.1439209 (PMC11333251; doi:10.3389/fonc.2024.1439209)
Supplement: Supplementary file 1 [file Table_1.docx]

Supplementary Material

Supplementary table 1 Top 10 cited references in studies of BMLC

| **Paper** | **DOI** | **Total Citations** | **TC per Year** | **Normalized TC** |
| --- | --- | --- | --- | --- |
| RIIHIMÄKI M, 2014, LUNG CANCER | 10.1016/j.lungcan.2014.07.020 | 528 | 48.00 | 12.86 |
| SAAD F, 2007, CANCER-AM CANCER SOC | 10.1002/cncr.22991 | 460 | 25.56 | 7.42 |
| SACHER AG, 2016, JAMA ONCOL | 10.1001/jamaoncol.2016.0173 | 441 | 49.00 | 19.63 |
| BROWN JE, 2005, JNCI-J NATL CANCER I | 10.1093/ji/dji002 | 437 | 21.85 | 5.58 |
| KATOH Y, 2009, CURR MOL MED | 10.2174/156652409789105570 | 429 | 26.81 | 7.79 |
| SHOLL LM, 2015, J THORAC ONCOL | 10.1097/JTO.0000000000000516 | 303 | 30.30 | 9.86 |
| WANING DL, 2015, NAT MED | 10.1038/nm.3961 | 257 | 25.70 | 8.36 |
| MIGLIORATI CA, 2006, LANCET ONCOL | 10.1016/S1470-2045(06)70726-4 | 247 | 13.00 | 4.76 |
| SCAGLIOTTI GV, 2012, J THORAC ONCOL | 10.1097/JTO.0b013e31826aec2b | 234 | 18.00 | 7.13 |
| LIPTON A, 2008, CANCER-AM CANCER SOC | 10.1002/cncr.23529 | 210 | 12.35 | 3.39 |

Supplementary table 2 Top 10 Keywords by counts on BMLC.

| Count | Centrality | Year | keyword |
| --- | --- | --- | --- |
| 277 | 0.16 | 2004 | lung cancer |
| 268 | 0.27 | 2004 | bone metastasis |
| 196 | 0.12 | 2010 | survival |
| 187 | 0.13 | 2004 | bone metastases |
| 166 | 0.07 | 2004 | breast cancer |
| 144 | 0.08 | 2004 | zoledronic acid |
| 122 | 0.07 | 2006 | prostate cancer |
| 114 | 0.11 | 2005 | non-small cell lung cancer |
| 93 | 0.09 | 2004 | disease |
| 87 | 0.09 | 2009 | expression |

**
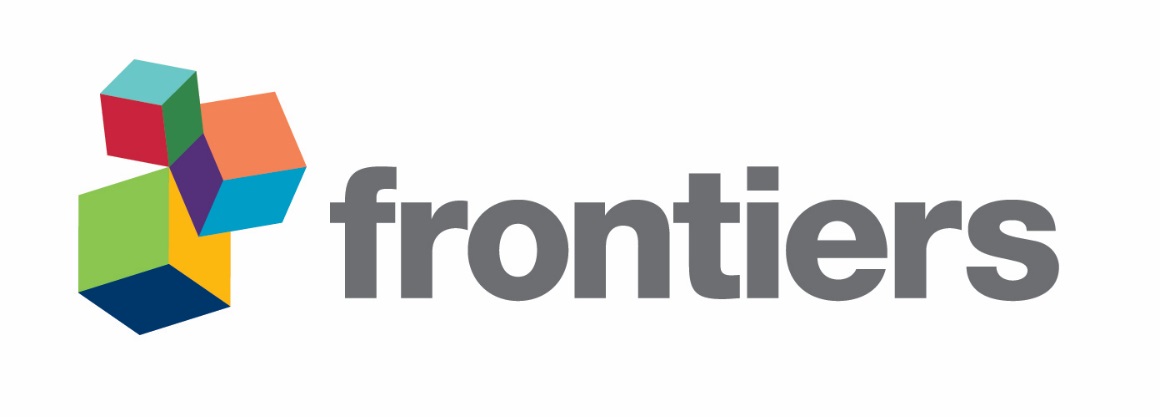
**
